# A Two-Stage Fitting Method for Truncated Stem Diameter Distributions

**DOI:** 10.1007/s44391-026-00069-5

**Published:** 2026-06-15

**Authors:** Gregory E. Paradis

**Affiliations:** Department of Forest Resources Management, University of British Columbia, Vancouver, Canada

**Keywords:** Truncated distributions, Fixed-area plots, Diameter modelling, Nonlinear optimisation, Reproducible research

## Abstract

Stem diameter distributions underpin forest planning and inventory practice worldwide, yet permanent sample plot inventories are routinely truncated by merchantability limits and diameter caps. Truncated densities are the standard remedy, but those forms are seldom documented or supported in common software, so practitioners default to biased complete-form fits. We introduce a two-stage weighted least-squares workflow that retains the familiar complete-form implementation while recovering truncated-fit accuracy. Stage one estimates a scaling factor alongside the density parameters; stage two freezes that normaliser to deliver unbiased shape and scale estimates. Applied to fixed-area plots from Québec, Canada, the two-stage estimator tracks truncated Weibull and gamma fits across 32 species-group/cover-type combinations (root-mean-square error between one-stage truncated and two-stage complete fits: $2\times 10^{-5}$–$3.7\times 10^{-2}$) and yields the lowest stage-2 AICc (small-sample Akaike information criterion) in 25 of 32 cases. The companion repository provides the tallies, scripts, and notebooks required to regenerate every figure, table, and LaTeX artefact.

## Introduction

Stem diameters in forest stands are commonly characterised by fitting statistical distributions to plot measurements (Bailey and Dell 1973; Hyink and Moser 1983; Gunn et al. 2000; Orellana et al. 2017; Orellana and Figueiredo Filho 2017). Because permanent sample plot (PSP) programmes are expensive to maintain, analysts often rely on legacy inventories whose tally rules prioritised field efficiency over statistical convenience.

Merchantability thresholds remove small stems, biological limits cap large diameters, and crews aggregate counts into fixed-width classes, leaving doubly truncated grouped stand tables.

Right truncation is less explicit in many inventories, so the upper bound should be interpreted as an operational or practical limit rather than a biological constant. In the PSP workflow we set *b* to the observed maximum (and matching management caps where available) to reflect how stand tables are used in practice; when no meaningful upper limit is defensible, analysts can treat the data as left-truncated only or introduce a location parameter in the candidate distribution. The two-stage estimator is agnostic to how *b* is chosen, but its interpretation depends on that choice.

Complete-form distributions defined on (0,∞) perform poorly on such data because tail bias persists and residual sums fail to vanish. Analysts therefore evaluate truncated versions of the target distribution (Zutter et al. 1986), yet closed forms are not always published, forcing teams to reproduce earlier derivations (Mittal and Dahiya 1989; Hannon and Dahiya 1999; Hegde and Dahiya 1989; Ahmad 2001; Wingo 1989) or numerically integrate $F(x; \boldsymbol{P}) = \int _0^x f(t)\,\textrm{d}t$.

We propose a two-stage method that retains the *complete* form while achieving truncated-quality fits. The method is benchmarked against one-stage complete (1sc) and one-stage truncated (1st) fits using permanent sample plot data from Québec, Canada. Across Weibull and gamma families the two-stage complete (2sc) estimator matches specialised truncated fits while preserving implementation simplicity.

## Methods

### Inventory Binning

The workflow operates on fixed-area permanent sample plots from the Québec provincial inventory. For each species-group / cover-type combination we aggregate tallies into 2 cm classes spanning 10–60 cm DBH (diameter at breast height). Each 0.04 ha plot receives the constant expansion factor $s_{\text {plot}} = 25$, yielding a stand table $\hat{y}_i = s_{\text {plot}} t_i$ for bin *i*, where $t_i$ is the mean tally across plots. We then normalise the stand table so that $\sum _{i \in I} W \hat{y}_i^{(n)} = 1$ for bin width W=2 cm, producing a truncated empirical density on [a,b]=[10,60] cm. The upper bound *b* reflects the observed maximum in the PSP tallies (and management caps where applicable); if no defensible upper limit exists, the workflow can be applied with a larger *b* or with left truncation only.

### Weighted Non-Linear Least Squares

We fit parametric distributions by matching the empirical truncated density with candidate PDFs at the bin midpoints using weighted non-linear least squares. The objective is

1 $$\begin{aligned} Z = \min _{\boldsymbol{P}} \sum _{i \in I} e_i^2, \end{aligned}$$

with residuals

2 $$\begin{aligned} e_i = w_i \left[ f(x_i; \boldsymbol{P}) - \hat{y}_i^{(n)}\right] , \end{aligned}$$

where $x_i$ is the class midpoint, *f* the density evaluated at $x_i$, and $\boldsymbol{P}$ the free parameters. We retain unit bin weights ($w_i = 1$) to streamline the narrative and align the comparison with the published truncated baseline. The companion repository notebook documents the plot-level tallies and demonstrates how to construct inverse-variance weights when analysts wish to down-weight sparse edge bins. Optimisation uses the Levenberg–Marquardt algorithm as implemented in lmfit (Levenberg 1944; Marquardt 1963).

Truncated datasets exhibit biased residual sums when complete-form densities are fit directly. The recommended approach rescales the target density to produce the truncated form

3 $$\begin{aligned} f^{T}(x; \boldsymbol{P}) = {\left\{ \begin{array}{ll} c\, f(x; \boldsymbol{P}) & a \le x \le b, \\ 0 & \text {otherwise}, \end{array}\right. } \end{aligned}$$

with scaling constant $c = [F(b; \boldsymbol{P}) - F(a; \boldsymbol{P})]^{-1}$ and *F* the cumulative distribution function. When a closed form for *F* is not available we evaluate the integral numerically.

### Two-Stage Estimator

The two-stage estimator includes a positive scaling parameter *s* so the complete-form fit honours the truncated support. Stage one solves

4 $$\begin{aligned} \min _{\boldsymbol{P},\, s>0} \sum _{i \in I} \left[ s f(x_i; \boldsymbol{P}) - \hat{y}_i^{(n)}\right] ^2, \end{aligned}$$

returning provisional parameters $\boldsymbol{P}^{(1)}$ and $s^{(1)}$. Stage two holds $s^{(1)}$ fixed, refits $\boldsymbol{P}$ with the same objective, and yields the final estimates $\boldsymbol{P}^{(2)}$. Because *f* integrates to one, the fitted scaling parameter provides an implied retained mass on [*a*, *b*] via $F(b;\boldsymbol{P})-F(a;\boldsymbol{P}) \approx 1/s$; in the limit of a perfect match to the truncated form, *s* equals the normalising constant *c*.

### Comparative Fitting Workflow

We compare three fitting strategies for each meta-plot:

1. **1sc** – a complete-form fit with the scaling fixed to s=1;
2. **1st** – a truncated likelihood fit using $f^{T}(x; \boldsymbol{P})$ on [*a*, *b*];
3. **2sc** – the two-stage complete-form procedure described above.

The 1sc configuration is retained intentionally as an operational baseline: in many applied inventory workflows, analysts still fit complete-form densities directly to truncated grouped data with no explicit truncation correction. Including 1sc therefore quantifies the practical consequence of omitting truncation handling, while 1st serves as the reference truncated fit and 2sc as the proposed complete-form alternative. We wrap each probability density function into an lmfit Model, initialise parameters from empirical moments, and minimise *Z* for all three configurations.

### Candidate Distributions and Model Selection

We concentrate on the two-parameter Weibull and gamma distributions, both specialisations of the generalised gamma family that cover common stand structures with few parameters. Each species-group / cover-type meta-plot is fit with both distributions under the 1sc, 1st, and 2sc regimes. We compute the small-sample Akaike information criterion,

5 $$\begin{aligned} \text {AICc} = \text {AIC} + \frac{2K(K+1)}{n - K - 1}, \end{aligned}$$

where *n* is the number of non-zero bins and *K* counts the number of free parameters plus the implicit error variance term. We adjust *K* to account for the additional scaling terms associated with each configuration: $K_{1sc} = |\boldsymbol{P}| + 1$, $K_{1st} = |\boldsymbol{P}| + 3$ (two truncation bounds plus variance), and $K_{2sc} = |\boldsymbol{P}| + 2$. The best model for each meta-plot is selected by minimising AICc. We report parameter estimates, associated standard errors, and AICc values for both stage-one and stage-two fits to facilitate comparison with the truncated baseline.

### Robustness Assessment Workflow

The robustness assessment has two components. First, all 32 species-group / cover-type combinations are fit and empirical histograms are classified into shape classes using a deterministic peak-location rule after light smoothing: inverse-J-like (peak in the first 20% of bins), mid-unimodal (20–60%), and right-peaked/flat (>60%). Method win counts (AICc), 1st–2sc discrepancy metrics, and fit-failure counts are then summarised by shape class.

Second, a canonical synthetic benchmark is evaluated with four scenario families (inverse-J, mid-unimodal, near-flat, and right-shifted) under fixed random seeds and replicated sample-size regimes. For each synthetic dataset, the same 1sc/1st/2sc workflow is run and win rates and discrepancy metrics are aggregated. Compact robustness summaries are provided in Appendix Tables 2–4.

**Fig. 1:**
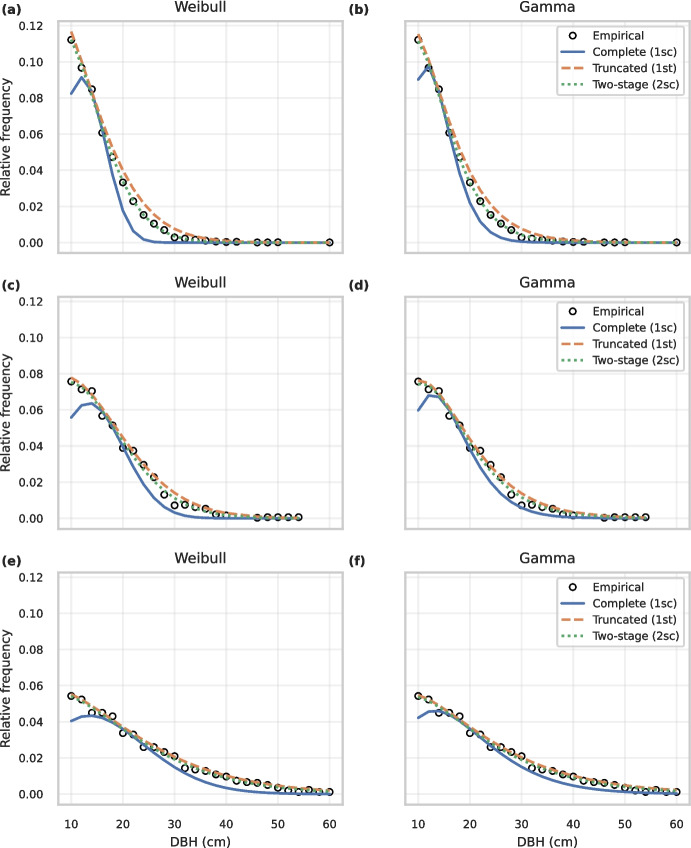
Comparison of Weibull (left column) and Gamma (right column) fits across three representative meta-plots: (**a**–**b**) SPFL softwood, (**c**–**d**) Birch mixedwood, and (**e**–**f**) Maple hardwood. Points denote empirical relative frequencies; solid lines show the one-stage complete fits, dashed lines the truncated fits, and dotted lines the two-stage complete fits

## Results

Figure 1 compares empirical histograms with fitted Weibull and gamma curves for three representative meta-plots (rows: SPFL-S, Birch-M, Maple-H; columns: Weibull, Gamma). Each panel shows the one-stage complete (1sc), one-stage truncated (1st), and two-stage complete (2sc) solutions. The truncated and two-stage curves remain close across the diameter range, while the single-stage complete fits deviate in the lower and central classes.

Table 1 summarises parameter estimates, AICc scores, and diagnostics comparing 1st to 2sc. The 2sc stage 2 AICc is lowest in 25 of 32 combinations, while the remaining seven cases slightly favour 1sc; 1st is never best. The RMSE between 1st and 2sc fits ranges from $2\times 10^{-5}$ to $3.7\times 10^{-2}$, and the 1st fit fails in two cases (CHCE–R and ERS–R), producing infinite AICc, whereas the 2sc workflow still converges.

**Table 1:** Parameter estimates (stage 1/2), AICc scores, and 1st–2sc diagnostics (RMSE, max abs diff) for each species-group / cover-type combination.

Species	Cover		Stage 1	Stage 2	AICc	AICc	AICc	AICc	RMSE	
Group	Type	Distribution	Parameters	Parameters	(1sc)	(1st)	(2sc S1)	(2sc S2)	(1st–2sc)	Max |1st–2sc|
auf	f	weibull	a = 0.12±0.00, beta = 0.00±0.00	a = 0.12±0.00, beta = 0.00±0.00	-206.863	-242.565	-245.644	-247.644	0.002144	0.003064
auf	m	gamma	beta = 10.90±4.35, p = 0.71±0.59	beta = 10.90±1.13, p = 0.71±0.05	-169.411	-189.871	-192.217	-194.217	0.001913	0.002508
auf	r	gamma	beta = 0.37±0.07, p = 33.14±6.38	beta = 0.37±0.05, p = 33.14±4.47	-70.647	-46.670	-64.192	-66.192	0.017329	0.033565
boj	f	gamma	beta = 24.72±9.99, p = 0.92±0.36	beta = 24.72±2.67, p = 0.92±0.08	-247.033	-263.565	-263.393	-265.393	0.000720	0.000816
boj	m	weibull	a = 1.02±0.14, beta = 21.50±1.55	a = 1.02±0.04, beta = 21.50±1.46	-258.194	-284.908	-285.645	-287.645	0.000896	0.001063
boj	r	gamma	beta = 8.18±3.71, p = 2.37±1.05	beta = 8.18±1.55, p = 2.37±0.45	-177.887	-178.028	-177.723	-179.723	0.000605	0.000836
bop	f	weibull	a = 2.28±0.08, beta = 18.22±0.22	a = 2.28±0.05, beta = 18.22±0.21	-209.283	-225.171	-234.853	-236.853	0.001860	0.002903
bop	m	weibull	a = 1.77±0.07, beta = 15.52±0.25	a = 1.77±0.03, beta = 15.52±0.21	-203.165	-238.620	-253.311	-255.311	0.002018	0.003136
bop	r	gamma	beta = 5.69±1.85, p = 1.70±0.85	beta = 5.69±0.50, p = 1.70±0.12	-139.248	-151.230	-156.300	-158.300	0.003317	0.004613
chce	f	weibull	a = 3.42±0.28, beta = 27.09±0.68	a = 3.42±0.23, beta = 27.09±0.65	-236.226	-233.005	-233.798	-235.798	0.001993	0.003690
chce	m	weibull	a = 1.13±0.43, beta = 28.25±7.11	a = 1.13±0.12, beta = 28.25±4.34	-167.925	-171.790	-172.133	-174.133	0.001499	0.002327
chce	r	weibull	a = 0.08±0.00, beta = 0.00±0.00	a = 0.08±0.00, beta = 0.00±0.00	-18.081	∞	1.627	-0.373	0.029990	0.035010
ers	f	weibull	a = 1.35±0.05, beta = 19.09±0.34	a = 1.35±0.02, beta = 19.09±0.30	-270.662	-322.987	-334.847	-336.847	0.001089	0.001399
ers	m	weibull	a = 1.66±0.12, beta = 22.22±0.73	a = 1.66±0.07, beta = 22.22±0.71	-234.636	-250.082	-249.851	-251.851	0.000514	0.000655
ers	r	gamma	beta = 5.52±13.71, p = 1.46±6.16	beta = 5.52±2.41, p = 1.46±0.48	-24.338	∞	1.662	-0.338	0.004109	0.005794
erx	f	weibull	a = 1.69±0.14, beta = 16.63±0.60	a = 1.69±0.06, beta = 16.63±0.52	-200.812	-219.942	-222.243	-224.243	0.001539	0.002214
erx	m	gamma	beta = 3.53±0.18, p = 4.21±0.23	beta = 3.53±0.08, p = 4.21±0.10	-244.189	-264.003	-289.084	-291.084	0.002249	0.003869
erx	r	gamma	beta = 3.03±1.11, p = 3.39±1.64	beta = 3.03±0.29, p = 3.39±0.28	-98.018	-99.619	-106.439	-108.439	0.005386	0.008685
peu	f	weibull	a = 2.49±0.24, beta = 29.46±1.18	a = 2.49±0.19, beta = 29.46±1.09	-233.966	-232.663	-231.893	-233.893	0.000065	0.000082
peu	m	weibull	a = 2.95±0.13, beta = 26.76±0.41	a = 2.95±0.10, beta = 26.76±0.39	-265.655	-263.215	-263.299	-265.299	0.000677	0.000991
peu	r	gamma	beta = 4.26±1.30, p = 5.43±1.49	beta = 4.26±0.91, p = 5.43±1.08	-159.294	-156.320	-156.243	-158.243	0.000023	0.000032
pib	f	gamma	beta = 7.80±5.91, p = 1.21±1.49	beta = 7.80±1.52, p = 1.21±0.17	-124.811	-125.730	-126.867	-128.867	0.002379	0.003429
			beta = 3457886.22±362415627350.15,	beta = 3458187.07±10130587.09,						
pib	m	gamma	p = 0.70±0.86	p = 0.70±0.19	-224.884	-231.926	-231.217	-233.217	0.000877	0.000915
pib	r	weibull	a = 2.37±0.22, beta = 36.04±1.57	a = 2.37±0.18, beta = 36.04±1.40	-260.683	-259.664	-258.812	-260.812	0.000268	0.000295
pir	m	weibull	a = 0.78±1.57, beta = 4470.31±749347.73	a = 0.78±0.17, beta = 4470.31±3896.35	-49.856	-37.249	-60.971	-62.971	0.030188	0.035307
pir	r	gamma	beta = 16.41±18.54, p = 3.00±2.12	beta = 16.41±5.28, p = 3.00±0.80	-41.074	-17.144	-42.376	-44.376	0.036560	0.060268
sepm	f	gamma	beta = 5.11±1.23, p = 1.41±0.66	beta = 5.11±0.30, p = 1.41±0.07	-178.725	-198.773	-214.144	-216.144	0.004236	0.006626
sepm	m	weibull	a = 1.08±0.06, beta = 9.15±0.56	a = 1.08±0.01, beta = 9.15±0.11	-215.363	-275.376	-300.992	-302.992	0.002202	0.003248
sepm	r	gamma	beta = 3.09±0.13, p = 3.92±0.19	beta = 3.09±0.04, p = 3.92±0.05	-203.145	-228.396	-277.128	-279.128	0.003404	0.006085
topu	f	weibull	a = 0.66±0.53, beta = 3.83±7.43	a = 0.66±0.07, beta = 3.83±0.31	-151.850	-155.087	-155.851	-157.851	0.002662	0.003482
topu	m	weibull	a = 1.21±0.12, beta = 16.80±0.97	a = 1.21±0.04, beta = 16.80±0.73	-255.424	-285.123	-287.392	-289.392	0.001222	0.001572
topu	r	gamma	beta = 5.24±0.51, p = 4.22±0.38	beta = 5.24±0.35, p = 4.22±0.27	-289.020	-291.630	-291.610	-293.610	0.000582	0.000838

Empirical shape-stratified summaries confirm that inverse-J profiles dominate the current PSP-derived data, but non-inverse-J classes are present and informative. Appendix Tables 2 and 3 report the shape-stratified win counts and discrepancy/failure summaries, showing that method rankings are not perfectly uniform across shape classes and reinforcing the need for bounded interpretation.

In the canonical simulation suite, 2sc and 1st remain close in most replicates, with disagreement concentrated in low-information scenarios (near-flat and right-shifted) and lower sample-size settings. Appendix Table 4 reports per-scenario win counts and aggregate discrepancy metrics. Together with the empirical summaries, these synthetic checks preserve direct comparability within the same 1sc/1st/2sc framework.

## Discussion

Across the meta-plots the two-stage estimator reproduced the truncated fits and most often delivered the lowest AICc among the three alternatives. The complete single-stage estimator remained biased near the truncation limits, whereas the truncated single-stage (1st) and two-stage complete (2sc) solutions were visually similar in Fig. 1. These findings show that the two-stage workflow typically matches the reference truncated procedure without bespoke truncated-density implementations, while acknowledging a handful of cases where 1sc is marginally preferred by AICc. We emphasize that 1sc is included as a deliberate operational benchmark rather than a strawman comparator: it reflects the common complete-form fitting pattern applied to truncated grouped stand tables when truncation is not modelled explicitly.

Two meta-plots (white pine–mixedwood and red pine–mixedwood) produce unusually large scale parameters in Table 1. Their truncated histograms are nearly flat, so the optimiser pushes shape parameters below one and inflates the scale to hold the area to unity. Both 1st and 2sc converge on the same regime, indicating a data-driven effect rather than a two-stage weakness.

We limited the candidate set to the two-parameter Weibull and Gamma distributions because they cover common stand structures with few parameters, both arise as special cases of the generalised gamma family, and a compact family keeps attention on the methodological swap between the truncated and two-stage estimators. A three-parameter Weibull (with location) is an alternative way to handle left truncation; we treat it as future work because it changes the parameterisation and increases flexibility in ways that would blur the direct 1st-vs-2sc comparison in a brief format.

A companion repository (see Data Availability statement for details) documents the tallies, weighting choices, and scripts that regenerate the analysis. Robustness summary tables are provided in the manuscript appendix (Tables 2–4). With that scaffold, 2sc matches or improves on the truncated baseline and outperforms the unadjusted 1sc baseline in most tested settings. Because the interpretation of *b* is application-specific, we view the two-stage workflow as an alternative that should be stress-tested under other truncation rules or distributional shapes before being treated as a universal replacement. The current analysis focuses on inverse-J PSP stand tables; other shapes warrant future study.

The robustness analysis includes empirical shape-stratified summaries across all 32 PSP combinations and a canonical synthetic benchmark spanning inverse-J, unimodal-mid, near-flat, and right-shifted cases. This evidence improves coverage of plausible stand-structure conditions, but it also confirms that method preference is context-dependent in low-information regimes. Accordingly, we frame 2sc as a strong practical option under tested conditions rather than a universal replacement.

## Conclusion

Fitting complete-form distributions to truncated inventory data yields biased estimates. The truncated-likelihood baseline is accurate but requires bespoke truncated PDFs for each candidate family. Our two-stage approach preserves complete-form simplicity while matching truncated fits. In the computational experiment it matched or exceeded the truncated baseline and dominated the naive complete-form approach.

The workflow automates data prep, fitting, and manuscript builds; code and anonymised intermediates accompany the manuscript for reuse.

## Data Availability

Scripts, processed datasets, and the companion notebook are available at https://github.com/UBC-FRESH/dbhdistfit-papers; raw PSP tallies originate from Données Québec and are accessible via its public portal.

## Appendix A: Derivations Supporting the Two-Stage Estimator

We briefly restate the derivations that motivate the two-stage fitting procedure. Let $f(x; \boldsymbol{P})$ denote a complete-form probability density defined on (0,∞) with parameter vector $\boldsymbol{P}$. Observations are only available for the truncated interval [*a*, *b*], with a=10 cm and b=60 cm in the PSP experiment. The truncated form of *f* is

6 $$\begin{aligned} f^{T}(x; \boldsymbol{P}) = {\left\{ \begin{array}{ll} c \, f(x; \boldsymbol{P}), & a \le x \le b, \\ 0, & \text {otherwise}, \end{array}\right. } \end{aligned}$$

where the normalising constant is

7 $$\begin{aligned} c = \left[ \int _a^b f(t; \boldsymbol{P}) \,\textrm{d}t\right] ^{-1} = \left[ F(b; \boldsymbol{P}) - F(a; \boldsymbol{P})\right] ^{-1}. \end{aligned}$$

Here *F* is the cumulative distribution function associated with *f*. When a closed-form expression for *F* is unavailable, *c* must be evaluated numerically, complicating routine model comparison.

To avoid deriving bespoke truncated forms for each target distribution, we augment the complete density with a free scaling parameter *s* and define

8 $$\begin{aligned} f'(x; \boldsymbol{P}', s) = s \, f(x; \boldsymbol{P}), \end{aligned}$$

where $\boldsymbol{P}' = \boldsymbol{P} \cup \{s\}$. Because *s* relaxes the unity constraint on the integral of *f*, the least-squares objective

9 $$\begin{aligned} Z(\boldsymbol{P}', s) = \sum _{i \in I} \left[ f'(x_i; \boldsymbol{P}', s) - \hat{y}_i^{(n)}\right] ^2 \end{aligned}$$

admits solutions with residual behaviour comparable to $f^{T}$ over [*a*, *b*]. The augmented model reproduces any truncated shape achievable by $f^{T}$ but introduces an additional degree of freedom that inflates parameter variance.

If the truncated solution is matched exactly on [*a*, *b*], then $f'(x)=f^{T}(x)$ implies $s=c=[F(b;\boldsymbol{P})-F(a;\boldsymbol{P})]^{-1}$. Hence 1/*s* is the implied retained mass on [*a*, *b*], and departures from that value quantify any residual mass assigned outside the truncation interval.

We therefore fit the model in two stages. Stage 1 solves

10 $$\begin{aligned} (\widehat{\boldsymbol{P}'}, \hat{s}) = \arg \min _{\boldsymbol{P}', s} Z(\boldsymbol{P}', s), \end{aligned}$$

yielding a consistent estimate $\hat{s}$ that scales the complete-form density to the truncated support. Stage 2 fixes the scaling parameter and re-optimises the original parameter vector,

11 $$\begin{aligned} \widehat{\boldsymbol{P}} = \arg \min _{\boldsymbol{P}} Z(\boldsymbol{P}, \hat{s}), \end{aligned}$$

where $Z(\boldsymbol{P}, \hat{s})$ denotes the objective with *s* held constant. The fitted curves coincide,

12 $$\begin{aligned} Z(\widehat{\boldsymbol{P}'}, \hat{s}) \simeq Z(\widehat{\boldsymbol{P}}, \hat{s}), \end{aligned}$$

but the second-stage covariance matrix excludes the nuisance scaling parameter, yielding tighter standard errors for the distributional parameters of interest.

The approach generalises to any probability density for which numerical quadrature of *f* over [*a*, *b*] is feasible.

## Appendix B: Robustness Summary Tables for Review

This appendix provides compact robustness summaries supporting the main-text method comparison. Reproducibility scripts and detailed machine-readable outputs are available in the companion repository (see Data Availability statement for details).

**Table 2:** Shape-stratified method-win counts across the 32 empirical species-group / cover-type combinations

Shape class	Winner method	Win count
inverse-J-ish	1sc	2
inverse-J-ish	2sc	25
right-peaked/flat	2sc	1
unimodal-mid	1sc	3
unimodal-mid	2sc	1

**Table 3:** Shape-stratified discrepancy and fit-failure summaries

	N	Mean RMSE	Mean max	Failures	Failures	Failures
Shape class	groups	(1st vs 2sc)	abs diff	1sc	1st	2sc
inverse-J-ish	27	0.004792	0.007224	0	0	0
right-peaked/flat	1	0.03844	0.05581	0	0	0
unimodal-mid	4	0.000491	0.0006817	0	0	0

**Table 4:** Canonical simulation scenario win counts and aggregate discrepancy metrics by sample size

	Sample	1sc	2sc	Mean RMSE	Mean max
Scenario	size	wins	wins	(1st vs 2sc)	abs diff
inverse-J	1500	0	40	0.0000622	0.0000727
inverse-J	4000	0	40	0.0000395	0.0000457
near-flat	1500	0	40	0.00000403	0.00000937
near-flat	4000	0	40	0.00000452	0.0000109
right-shifted	1500	39	1	0.0001708	0.0002829
right-shifted	4000	40	0	0.0001507	0.0002510
unimodal-mid	1500	7	33	0.0002970	0.0003730
unimodal-mid	4000	0	40	0.0002747	0.0003405

## Declarations

### Employment

G.E. Paradis is employed as an Assistant Professor at the University of British Columbia.

### LLM Use

LLM-assisted editing improved readability; the author approved all content.
